# Fever in Pregnancy: A Rare Case of Listeria-Induced Chorioamnionitis

**DOI:** 10.7759/cureus.70670

**Published:** 2024-10-02

**Authors:** Sousan Alhomsi, Lina Sawallha, Mohammad Hakmi, Ali Al Ibrahim, Hana Khayoun, Nausheen Henna

**Affiliations:** 1 Obstetrics and Gynaecology, Tawam Hospital, Al Ain, ARE; 2 Maternal-Fetal Medicine, Kanad Hospital, Al Ain, ARE; 3 Anatomic Pathology, Tawam Hospital, Al Ain, ARE

**Keywords:** antibiotic therapy, chorioamnionitis, fever in pregnancy, listeria in pregnancy, maternal infection, preterm delivery

## Abstract

Listeriosis is a rare infection during pregnancy and may result in complications such as chorioamnionitis. Diagnosis is hardly made due to the nonspecific symptoms of listeriosis. A 27-year-old pregnant woman at 27 weeks and 3 days of gestation was admitted with a history of fever for 6 days and decreased fetal movement. Administration of broad-spectrum antibiotics initially did not improve her condition. Amniocentesis was suggestive of chorioamnionitis, and culture was positive for *Listeria monocytogenes*. In view of that, delivery was indicated for the safety of the mother and baby. Her case highlights the necessity of maintaining a high index of suspicion for listeriosis in pregnant patients with a history of exposure who present with fever without an evident underlying cause. Early diagnosis and management are very important for a better prognosis in such an illness. Educating pregnant women about dietary precautions and prompt medical care may prevent severe complications.

## Introduction

Listeriosis is a rare foodborne disease. It is caused by gram-positive, facultative intracellular rod bacteria [1]. It can survive a wide range of temperatures (0-45 ^◦^C), PH (4.5-9), and high-salt environments (20% sodium chloride (NaCl)) [2]. These facts indicate that *Listeria* can be isolated from various foods, including deli-style meats and poultry, unwashed vegetables or store-made salads, unpasteurized milk, and smoked seafood [3].

The population at highest risk includes the elderly, immunocompromised individuals, pregnant women, and fetuses or neonates who have increased vulnerability and are prone to severe disease [4]. The incidence of *Listeria *in pregnant women is approximately 43% of total cases and 14% in late pregnancies [5]. Overall, pregnant women have an 18-fold increased risk of acquiring listeriosis infection than other healthy adults.

To reach a diagnosis of listeriosis in pregnancy is still challenging, as patients with *Listeria* infection are usually asymptomatic. However, it can manifest with flu-like symptoms, gastrointestinal problems, or even neurological complications, such as meningitis or brain abscesses, and in some cases, progresses to sepsis [6].

Infection can affect both mother and fetus. Neonates contract listeriosis via vertical transmission from the mother to the fetus. Infants born at an earlier gestational age have the highest mortality rate [7]. Evidence suggests that fetal and neonatal mortality is between 20% and 50% [8]. In addition, a retrospective study found that mortality rates were higher in neonates with low birth weight, born earlier than 28 weeks of gestation, with an Apgar score of less than 5 at 5 minutes [9]. Therefore, early detection and diagnosis, with prompt intervention, is essential to optimize the outcome.

## Case presentation

A 27-year-old woman, Gravida 4, Para 2 + 1, at 27 weeks + 3 days gestation, IVF pregnancy with pre-implantation genetic testing for aneuploidy (PGT-A), presented with a six-day history of fever associated with two days of decreased fetal movements and no other associated symptoms. The patient denied a history of vaginal bleeding or amniotic fluid leakage. She is a known case of beta-thalassemia trait. Regarding surgical history, she only had an umbilical hernia repair; otherwise, no significant history.

Upon admission, the patient’s vitals were stable except for having a fever (39.4 °C) and generalized body pain. Physical examination only revealed mild abdominal tenderness. Per speculum examination was unremarkable, with no significant findings. Initial laboratory results are summarized in Table 1.

**Table 1:** Initial laboratory results

Test	Patient Result	Reference Range	Units
White Blood Cells (WBC)	8.19	4.0 – 10.0	K/μL
Hemoglobin (Hb)	9.0	12.0 – 15.0	g/dL
Procalcitonin	0.06	Adults & Children ≥72 hours: ≤0.15	ng/mL
Lactic Acid	0.7	0.06 – 1.80	mmol/L
C-Reactive Protein (CRP)	69.12	≤5.0	mg/L

Urinalysis was negative and ultrasound showed normal fetal growth (29th percentile) with no signs of fetal anemia. The biophysical score was 2/8, and computerized cardiotocography (CTG) showed poor variability. Initially, the patient was started on empirical antibiotics, including IV meropenem, clindamycin, and gentamycin. Treatment was changed to IV ampicillin 1 g every six hours upon blood culture results that came back positive for *Enterococcus faecalis*.

Despite the treatment, the patient was not improving and continued to have intermittent fever with no clear focus for infection. A decision was made to perform amniocentesis on day 5 of admission. The initial result revealed intact membrane chorioamnionitis; the amniotic fluid was amber and turbid. Laboratory analysis showed a white blood cell count (WBC) of >2000 cells per cubic mm (normal range: 0-5 cells/mm³), and a glucose level of <11 mg/dL (normal range: 40-70 mg/dL) with few pus cells noted. The patient was transferred to a tertiary hospital with neonatal intensive care unit (NICU) facilities to plan delivery accordingly.

Upon arrival at the tertiary hospital, the patient was started on magnesium sulfate (MgSO4), received two doses of betamethasone, and was booked for a category 3 cesarean section. Surgery was performed without any complications with an estimated blood loss of 500 ml, and the placenta was sent for histopathology. The baby's weight was 950 g with Apgar 4, 7, and 8 at 1, 5, and 10 minutes, respectively. The baby was admitted to the NICU for further management, and the mother had an uneventful recovery. Post-delivery amniotic fluid culture revealed *Listeria monocytogenes*, antibiotics were updated to piperacillin-tazobactam 4 gm q8hr for 5 days.

**Figure 1:**
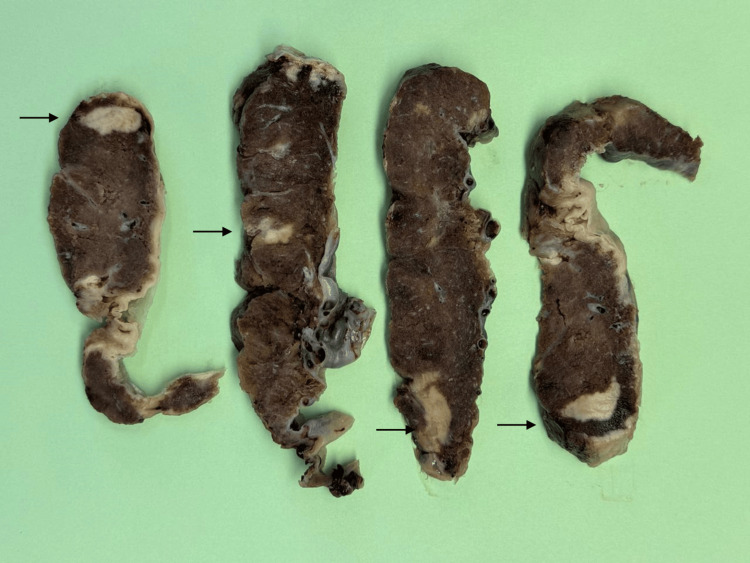
Gross placental examination in listerial infection Gross examination of the placental disc showing macro abscesses (arrows), as confirmed by microscopy.

**Figure 2:**
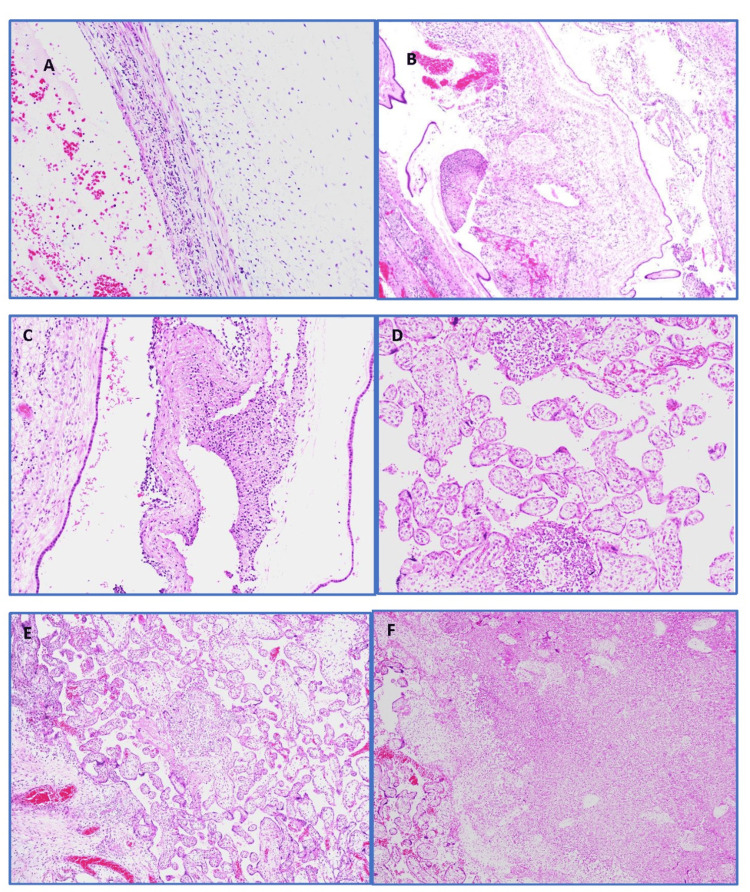
Histopathological findings in listerial placentitis A (×10): Histological examination of umbilical cord vein vasculitis; B & C (×100): Acute chorionitis with microabscess formation; D & E (×10): Acute villitis with intervillous microabscess formation; F (×200): Areas of villous necrosis with microabscess formation

Placental histopathology showed histological features suggestive of *Listerial placentitis*. Membranes revealed acute chorioamnionitis and fetal vascular involvement. Placental parenchyma showed features of acute villitis and intervillositis with macro and microabscesses and villous necrosis (Figure 1 and Figure 2).

Antibiotics were downgraded to ampicillin 2 g q6hrs and ceftriaxone 2 g q24hrs for a total of 14 days. The patient recovered well and was discharged from the hospital. The baby continued to be under NICU care till the issuing date of this report.

## Discussion

Due to non-specific symptoms, it is often hard to reach a diagnosis of listeriosis, as 30% of affected women are asymptomatic, which can be misleading [6]. In our case, the only symptom was persistent fever, which can be attributed to multiple illnesses. A high index of suspicion combined with accurate medical history and exclusion of more common causes is crucial for reaching the correct diagnosis.

One of the notable causes of preterm birth is chorioamnionitis, with the highest risk occurring in pregnancies <30 weeks of gestation [10]. It contributes to 40% to 70% of all premature births around the world. The reVITALize initiative outlines the definition of chorioamnionitis by having unexplained fever (>38 ºC or 100.4 ºF) with one or more of the following symptoms or signs: (1) uterine tenderness/irritability, (2) leukocytosis, (3) fetal tachycardia, (4) maternal tachycardia, and (5) malodorous vaginal discharge [11]. Chorioamnionitis can develop commonly (around 70%) due to an ascending polymicrobial infection from the genital area with *Ureaplasma species* being the most frequent microorganism. Chorioamnionitis through hematogenous spread is rarely identified as in the case with *Listeria monocytogenes* [11,12]. In our case, as no source of fever was found and the patient showed no improvement despite being on broad-spectrum antibiotics, this raised the idea of performing amniocentesis to rule out chorioamnionitis as the cause of pyrexia.

The gold standard for the diagnosis of listeriosis (maternal/fetal) is either blood or placental culture [6]. Culture of the placental tissue is the most sensitive in diagnosing maternal-neonatal listeriosis [13]. In our case, placental culture confirmed the diagnosis of *Listeria*.

Regarding the treatment of *Listeria* in pregnancy, a high dose of parenteral amoxicillin (>6 g/day) is recommended [6,13]. In cases of penicillin allergy, alternatives including trimethoprim-sulfamethoxazole (TMP-SMX) or erythromycin can be used while keeping in mind that TMP-SMX could have an adverse effect on the fetal heart and nervous system if given in the early stages of pregnancy. On the contrary, erythromycin concentration reduced after crossing the placenta, requiring an increase in the dose [13]. Generally, two to three weeks is a sufficient period of treatment, except if neurological involvement is present; then, the period is extended to four weeks [6]. Our patient was started on broad-spectrum antibiotics till diagnosis was confirmed, antibiotics were then adjusted accordingly.

Finally, there is a higher risk for infection with the consumption of several dairy products, including unpasteurized raw milk, soft cheeses, and Hispanic-style cheeses, as well as undercooked, raw, smoked, or processed meat, and ready-to-eat foods [14]. Educating and counseling women antenatally will significantly increase awareness and knowledge regarding safer dietary options, thus reducing similar conditions during pregnancy.

## Conclusions

*Listeria* is a rare and difficult disease to diagnose due to nonspecific symptoms and presentation. A history of high-risk diet and suspicion of flu-like illness in some pregnant women could raise the possibility of such a condition. Appropriate food preparation, handling, and storage prevent bacterial growth and contamination. Educating patients and raising awareness about better and safer dietary options during pregnancy would lead to a reduction in morbidity and mortality for both the baby and the mother. Rapid detection and management have been proven to improve maternal and fetal outcomes.
